# Metabolomics to Explore Impact of Dairy Intake

**DOI:** 10.3390/nu7064875

**Published:** 2015-06-17

**Authors:** Hong Zheng, Morten R. Clausen, Trine K. Dalsgaard, Hanne C. Bertram

**Affiliations:** 1 Department of Food Science, Aarhus University, Kirstinebjergvej 10, Aarslev DK-5792, Denmark; E-Mails: hong.zheng@food.au.dk (H.Z.); mortenr.clausen@food.au.dk (M.R.C.); 2 Department of Food Science, Aarhus University, Blichers Allé 20, Tjele DK-8830, Denmark; E-Mail: trine.dalsgaard@food.au.dk

**Keywords:** dairy product, casein, whey, cheese, biomarkers, compliance

## Abstract

Dairy products are an important component in the Western diet and represent a valuable source of nutrients for humans. However, a reliable dairy intake assessment in nutrition research is crucial to correctly elucidate the link between dairy intake and human health. Metabolomics is considered a potential tool for assessment of dietary intake instead of traditional methods, such as food frequency questionnaires, food records, and 24-h recalls. Metabolomics has been successfully applied to discriminate between consumption of different dairy products under different experimental conditions. Moreover, potential metabolites related to dairy intake were identified, although these metabolites need to be further validated in other intervention studies before they can be used as valid biomarkers of dairy consumption. Therefore, this review provides an overview of metabolomics for assessment of dairy intake in order to better clarify the role of dairy products in human nutrition and health.

## 1. Introduction

Cow’s milk is a good source of nutrients for humans and its health benefits have been appreciated since the Middle Ages [1]. Currently, cow’s milk plays an important role in human nutrition, spanning the entire age range from infants to elderly, especially in Western countries but also increasingly in Asia and Africa. Cow’s milk can be processed into different types of dairy products, such as butter, cheese, cream, and yogurt. Due to the different dairy processes, nutritional characteristics may differ among dairy products, which results in different impacts on human health [2]. Therefore, it is of great importance to qualitatively and quantitatively evaluate consumption of different dairy products in order to better correlate the effect of dairy intake to human health. Metabolomics aims at profiling all low-molecular-weight metabolites in a biological system and has been used as a promising tool to discriminate different dietary patterns [3], and also to identify dietary biomarkers [4]. Therefore, metabolomics could potentially be used as tool to assess dietary intake of specific food items in an unbiased way and thereby reduce human error and subjective bias, such as misremembering, under-reporting and misclassification of a specific diet, from traditional dietary assessment methods [5]. However, application of metabolomics for assessment of dairy intake is still in its infancy and solid markers for dairy intake still need to be identified and models that can quantify dairy intake based on analysis of bio fluids, such as urine, are still warranted.

Therefore, the main aim of this review is to emphasize state-of-the-art assessment of dairy intake using metabolomics and encourage its application in this area. The present review consists of (i) a short introduction of dairy processing and nutrients; (ii) a brief description on the association between dairy intake and human health; (iii) a mini review of the application of metabolomics for assessment of dairy consumption; and (iv) concluding remarks and future perspectives.

## 2. Dairy Products and Composition

**Figure 1:**
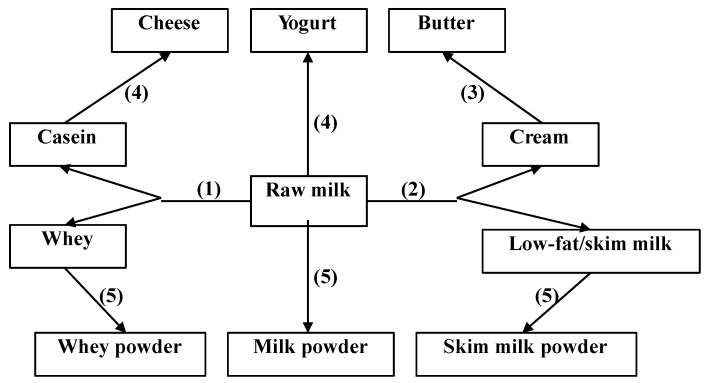
Overview of milk and various dairy products obtained from different processing schemes: (1) coagulation; (2) separation; (3) churning; (4) fermentation; (5) evaporation.

Cow’s milk is a liquid formed in the mammary glands of dairy cows during lactation and it is composed of water, protein (approximately 80% casein and 20% whey), fat (approximately 98% triglycerides), lactose, vitamins, and minerals [6]. Milk is often consumed after minimal processing including removal of varying amounts of fat and heat treatment, but milk is also to a large extent processed to other dairy products. Figure 1 displays an overview of the main dairy product categories. The milk fat can be concentrated from raw milk (4.0% fat) by centrifugation to obtain cream (up to 48% fat) and low-fat (1.0%–2.0% fat) or skim milk (< 0.5% fat) [6]. Thus, fat-soluble vitamins (e.g., vitamins A and D) in low-fat or skim milk are reduced or removed with the fat. In order to be compensated for this vitamin reduction, low-fat milk can be fortified with the fat-soluble vitamins, especially vitamin D. Butter is produced by churning cream to achieve a phase inversion from oil-in-water to water-in-oil emulsion [6]. In addition, fermented milk represents a large proportion of dairy products. Cheese, as a popular fermented product, is formed after coagulation of casein micelles using the enzyme chymosin, starter bacteria or heat treatment [7]. Numerous different varieties of cheese exist and their nutrients differ with the cheese type. In general, cheese consists of casein, saturated fat, fat-soluble vitamins, calcium, and bioactive peptides [7]. Yogurt is also a fermented dairy product obtained from a fermentation process by lactic acid bacteria, which produces lactic acid from lactose. The composition of yogurt is comparable to the original milk, while many nutrients in yogurt are more concentrated [8]. Whey protein can be concentrated as a by-product from cheese production and used as an additive in food to enhance specific physical properties or improve its nutritional value. Moreover, water can be removed from milk or other dairy products by evaporation to produce dry dairy products.

## 3. Dairy Intake and Human Health

Dairy products as nutrient-rich sources have been recommended as an important component in human nutrition and associated with potential health benefits.

### 3.1. Overweight and Obesity

Overweight and obesity are becoming more and more common health issues and their prevalence is rapidly growing in the world [9]. Many studies have examined the relationship between dairy consumption and body weight regulation. A systematic review based on prospective cohort studies revealed that the weight-lowering effect of dairy intake is suggestive but not consistent [10]. A meta-analysis from Abargouei *et al.* [11] reported that the effect of dairy intake on body weight depended on the energy intake; without energy restriction no effect of dairy intake was observed, but with energy restriction a beneficial effect of dairy intake on body weight was observed. Similarly, Chen *et al.* [12] found that dairy intake only attenuated body weight gain in the short-term or in energy-restricted studies, but not in the long-term or in non-energy-restricted studies. Dairy products have traditionally been considered to have an undesirable effect with respect to overweight and obesity due to a high content of saturated fat. Yet, in a recent systematic review the intake of high-fat dairy products was inversely linked with adiposity in 11 of 16 studies and adverse effects of high intake of dairy fat [13] could not be supported. Mechanisms explaining the potential weight-lowering effects of dairy intake have been suggested. The high calcium content in dairy products may facilitate a weight loss: firstly, calcium can suppress lipogenesis and stimulate lipolysis [14]; secondly, calcium can increase fecal fat excretion by combining with fatty acids and thereby forming insoluble soaps in the gut [15]; thirdly, calcium may increase fat oxidation [16]. Recently, milk and primary its medium chain fatty acids showed an up-regulating effect of the *ANGPTL4*-gene [17,18]. The *ANGPTL4*-gene increases the production of the ANGPTL4 protein, which has great impact on the uptake of fat from the blood stream to the adipose tissue by inhibition of lipoprotein lipase, but which recently also was shown to inhibit the pancreatic lipase in the gut [19], thus potentially also reducing the uptake of dietary fat. In addition, milk protein, especially whey, may regulate body weight: firstly, whey increases satiety and reduces energy intake [20]; secondly, whey enhances the thermic effect of food, resulting in higher post-meal energy expenditure [21]; thirdly, whey suppresses lipogenic enzyme in the adipose tissue [22]. In addition, fermented dairy products have been shown to result in increased gut bacterial content in humans [23], which must be considered an intriguing finding as there is increasing attention to the role of gut microbiota on the development of obesity [24].

### 3.2. Diabetes

Diabetes is a growing challenge for human health. Interestingly, an inverse correlation between dairy intake and the incidence of type 2 diabetes (T2D) has been established. In a meta-analysis performed by Pittas *et al.* [25], a lower T2D risk was found in the highest dairy intake group (3–5 servings/day) compared with the lowest dairy intake group (1.5 servings/day). Elwood *et al.* [26] also reported that the relative risk of T2D is almost 10% lower after high milk intake. Moreover, several meta-analyses found that low-fat or fermented dairy products protect against T2D [27,28,29,30]. Yet, a recent systematic review based on short- and long-term intervention studies did not show a consistent association between dairy intake and insulin sensitivity [31]. The mechanism behind the impact of dairy consumption on T2D can likely be explained partly by the weight-lowering effect of dairy intake (Section 3.1). In addition, several components in dairy products are suggested to protect against T2D. Dairy minerals, such as calcium and magnesium, have a potential for improving insulin sensitivity [32,33]. Intake of vitamin D has been associated with a lower risk of T2D by reducing insulin resistance [34]. Insulinotropic and glucose-lowering properties of whey protein have been reported in healthy subjects and patients with T2D [35]. Free fatty acids have been shown to increase insulin secretion from pancreatic β cells by activating G-protein-coupled receptor, GPR40 [36]. *trans*-Palmitoleate, primarily from dairy fat, may also reduce insulin resistance and incident diabetes [37].

### 3.3. Hypertension

Intake of milk and low-fat dairy products has been reported to hold an inverse correlation with hypertension risk [38,39]. A meta-analysis based on randomized controlled trials revealed that probiotic fermented milk protects against hypertension [40]. Several mechanisms behind the association between dairy intake and hypertension have been proposed. Firstly, the weight-lowering effect of dairy consumption is responsible for a lower risk of hypertension according to a meta-analysis performed by Neter *et al.* [41], where they found that systolic and diastolic blood pressure decreased by 1.1 and 0.9 mm Hg, respectively, with a decrease in body weight of 1 kg. Secondly, calcium intake from dairy products can maintain smooth muscle tone in blood vessels [42,43]. Other dairy minerals including magnesium [44], calcium [45], and potassium [46] may also contribute to a hypotensive effect. Thirdly, casein hydrolysates containing Val-Pro-Pro and Ile-Pro-Pro peptides have been observed to lower blood pressure in intervention studies [47,48,49], which is attributed to an inhibition of angiotensin converting enzyme (ACE). Several other possible peptide inhibitors of ACE have also been identified from milk, such as casokinins [50], C12 peptide [51], lactotripeptides [52], and lactokinins [53]. Moreover, milk peptides, such as lactokinin, may also regulate the release of endothelin-1, which can raise blood pressure by constricting blood vessels [54,55].

### 3.4. Cancer

Meta-analyses show an inverse correlation between dairy intake and colorectal cancer [56], a direct correlation between dairy ingestion and prostate cancer [57], while the correlations between dairy consumption and breast cancer [58], pancreatic cancer [59] and ovarian cancer [60] are inconclusive. For bladder cancer, the link varied with different types of dairy products [61,62]. The risk of cancer has been associated with obesity and T2D, which may be attributed to a high IGF-I level, insulin resistance and hyperinsulinemia [63,64]. Therefore, the effect of dairy intake on obesity and T2D may partly have contributed to the link between dairy consumption and cancer risk (Section 3.1 and Section 3.2). Yet, milk consumption may increase the IGF-I level, indicating an adverse effect of milk intake on cancer development [65]. An inadequate intake of calcium and vitamin D is associated with an increased risk of cancer [66]. Whey protein exerts anticancer properties by increasing the level of glutathione, which can reduce reactive oxygen species and carcinogens [67]. Bovine lactoferrin also plays a role in cancer prevention by induction of apoptosis and regulation of carcinogen-metabolizing enzymes [68]. Dairy fat like conjugated linoleic acid may also protect against cancer [69]. In addition, Lampe [70] suggested that the associations between fermented dairy products, gut microbiota, and cancer risk needs further exploration. Overall, due to the complexity of cancer, the mechanisms behind the effect of dairy intake on cancer risk remain unresolved.

### 3.5. Stroke

A recent meta-analysis revealed that low-fat or fermented dairy products were significantly related to low risk of stroke [71]. Moreover, a non-linear dose dependency was established between milk intake and relative stroke risk, in which the highest protective effect was found at approx. 200 mL/day and such an effect remained up to 700 mL/day. The correlation between dairy intake and stroke can most likely be attributed to the weight-lowering effect of dairy consumption [72]. Another possible mechanism might be that dairy consumption can reduce platelet aggregation and insulin resistance [73]. In addition, dairy minerals such as K, Ca, and Mg potentially contribute to reduce stroke risk [73].

### 3.6. Bone Health

Dairy product intake plays an important role in bone development during childhood and adolescence and prevention of bone loss in the elderly, which is attributed to the high contents of calcium, vitamin D, potassium, and phosphorus in dairy products [74,75].

## 4. Dairy Intake Assessment

### 4.1. Significance of Dairy Intake Assessment

Epidemiological evidence for a role of dairy products on human health has been comprehensively reported, yet their correlations differed with different types of dairy products, human diseases, and dietary regime (Section 3). A possible explanation for this discrepancy sometimes observed could be the different compositions of various dairy products. In addition, a poor or incorrect assessment of dairy intake may be another reason for this discrepancy. Thus, one of the greatest challenges for achieving a better understanding of dairy health in humans relies on the ability to obtain trustworthy information on dairy intake. Dietary assessment can be classified into two levels; qualitative and quantitative assessment. The former aims at discriminating between different diet groups, while the latter attempts to evaluate the amount of dietary intake. Food frequency questionnaires (FFQ), food records, and 24-h recalls are traditionally used to quantitatively assess dietary intake. However, these methods have several limitations, such as misremembering, under-reporting, and misclassification of the specific food items [76], which may mask important or make incorrect links between dietary intake and human health. Therefore, finding a reliable assessment method, such as biomarker utilization, is of great importance. Biomarkers, as measurable indicators of biological features, are categorized into exogenous and endogenous markers based on their origin [77]. Exogenous biomarkers can be applied to evaluate potential exposure to a specific diet, while endogenous markers are directly associated with metabolic changes in response to dietary ingestion. Thus, using exogenous biomarkers to evaluate dairy consumption may reduce the limitations of traditional assessment methods, improve the reliability and accuracy of dairy intake data, and thereby better elucidate the impact of dairy intake on human health.

### 4.2. Assessment of Dairy Fat Intake

By correlating the level of fatty acids in biological samples with the dietary data from traditional methods, several biomarkers for assessment of dairy fat intake have been identified. Wolk *et al.* [78] found that pentadecanoic (15:0) and heptadecanoic (17:0) acids in adipose tissue might be validated as exogenous biomarkers of long-term milk fat intake. The content of 15:0 in serum was also identified as a marker of dairy fat intake [79,80,81]. Wolk *et al.* [80] identified a new marker, myristic acid (14:0), in adipose tissue for dairy fat intake. Biong *et al.* [82] confirmed that the levels of 14:0, 15:0, and 17:0 in adipose tissue can be regarded as valid biomarkers of dairy fat intake. Moreover, 14:1 and 17:1 could be two new potential markers and serum 15:0 cholesteryl esters is the best alternative if adipose tissue is not obtainable [82]. Moreover, plasma and erythrocyte levels of 15:0 and *trans*-16:1n-7 might be indicators to assess dairy fat consumption [83]. 

Furthermore, there have been attempts to associate these biomarkers with human diseases for exploring the health benefits of dairy intake. A higher plasma level of 15:0 was linked to a higher risk of ischemic heart disease in women [83]. Warensjö *et al.* [84] established an inverse correlation between the level of milk fat biomarkers (15:0 and 17:0) in plasma and risk of developing a first myocardial infarction. Yet, the levels of 15:0 and 17:0 in adipose tissue were not associated with myocardial infarction risk, probably due to the influence of other nutrients [85]. The plasma *trans*-16:1n-7 level was inversely correlated with blood pressure and T2D incidence [37]. The content of 15:0 but not 14:0 and *trans*-16:1n-7 in plasma was observed to have an inverse correlation with cardiovascular disease and coronary heart disease [86]. Moreover, Santaren *et al.* [87] found that the serum 15:0 level was inversely correlated with T2D risk. Plasma 14:0, 15:0, 17:0, and *trans*-16:1n-7 levels were not significantly associated with stroke risk [88].

However, recently Ratnayake [89] raised concern in relation to the use of these fatty acids as biomarkers of dairy fat intake. This concern is related to the fact that the fatty acids 15:0, 17:0, and *trans*-16:1n-7 may also be derived from other food sources than dairy fat, and *trans*-16:1n-7 may also be endogenously synthesized from β-oxidation of dietary vaccenic acid. In addition, analytical challenges exist as these fatty acids need to be identified carefully due to small amounts in samples and co-elution with other fatty acids during GC analysis. Lankinen & Schwab [90] also suggested that 15:0 and 17:0 might be used as valid biomarkers of dairy fat intake if subjects consume a high amount of dairy fat and a low amount of fish. Overall, care must be taken to use these fatty acids in dietary assessment studies of dairy intake, and dietary origin and the analytical method should be taken into account.

Dairy fat in different matrices may possess different health effects. For instance, cheese fat lowers LDL-cholesterol level more than butter fat [91,92]. Therefore, the discrimination between consumption of different types of dairy products is also of great interest and important for elucidating the impact of dairy fat on human health.

### 4.3. Principle in Assessment of Dairy Intake by Metabolomics

**Figure 2:**
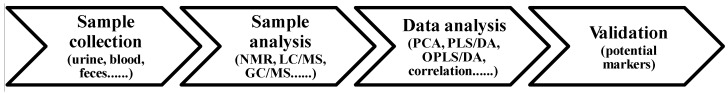
The basic procedure of metabolomics for dietary intake assessment.

Metabolomics aims to provide a comprehensive profile of all low-molecular-weight metabolites in the biological system under a particular condition, such as a dietary intervention. Metabolites can be regarded as exposure markers or metabolic products after consumption of a specific food. The basic procedure of metabolomics is shown in Figure 2: Firstly, biological samples typically urine, blood and/or feces are collected from subjects consuming a specific food; secondly, metabolite profiles of the samples are measured by analytical techniques such as nuclear magnetic resonance (NMR), liquid chromatography-mass spectrometry (LC-MS) and gas chromatography-mass spectrometry (GC-MS); thirdly, multivariate and univariate analyses are applied to analyze data in order to identify potential metabolites related to dietary intake; and finally, potential markers are validated in further studies. However, the validation of candidate biomarkers is rarely performed in most published metabolomics studies on dietary intake evaluation, which hampers the use of these biomarkers in clinical studies.

Metabolomics provides a good opportunity in dietary intervention studies to identify biomarkers for assessment of dietary intake [3,5]. Yet, metabolomics is also facing many challenges, such as metabolite identification and efficient data analysis of the comprehensive data sets [93]. Except technical challenges, intrinsic (e.g., genotype, gender, age, health status) and extrinsic (e.g., diet, drug, physical activity) factors that influence the metabolome also challenge the development of metabolomics in dietary nutrition studies [94].

### 4.4. Metabolomics Applied to Identify Biomarkers Related to Dairy Intake 

Metabolomics in combination with data analysis can potentially identify biomarkers, fingerprints, and associated models that can assess dairy intake quantitatively. However, only a few studies have applied metabolomics with main focus on the identification of biomarkers related to the intake of dairy products. In order to review current metabolomics studies on dairy products intake, we did a systematic literature search using the databases, PubMed [95], EMBASE [96] and SCOPUS [97], from 1995 to May 2015 by using the following search terms: (“dairy” OR “milk” OR “cheese” OR “butter” OR “casein” OR “whey”) AND (“metabolomic” OR “metabonomic” OR “metabolic profiling” OR “metabolite” OR “metabolomic” OR “mass spectrometry” OR “nuclear magnetic resonance” OR “LC MS” OR “GC MS” OR “NMR”) AND (“random” OR “andomly” OR “randomized” OR “control” OR “controlled” OR “cross over” OR “intervention” OR “trial”) AND (“urine” OR “urinary” OR “blood” OR “plasma” OR “serum” OR “feces” OR “faeces” OR “fecal”). The selected literature was published in English and conducted in human subjects.

Metabolomics was applied to identify dietary biomarkers in an epidemiologic study by Guertin *et al.* [98]. In this study, serum metabolites were measured using MS-based metabolomics and intake of 36 different dietary groups was recorded through the FFQ method. Using a correlation analysis between the MS-based metabolome and the FFQ data, Guertin *et al.* [98] found that serum 15:0, 16:0, and 10-undecenoate levels were directly correlated with butter consumption. However, the disadvantage of the FFQ such as under- or over-reporting of food consumption should be taken into account in this study.

### 4.5. Metabolomics Applied to Elucidate Metabolic Impact of Dairy Intake

Metabolomics has been applied for the assessment of both whole product and dairy protein intake under different experimental designs. Table 1 shows changes in metabolites detected as a function of dairy intake by metabolomics. These metabolites may be potential markers for quantitative assessment of dairy consumption. Metabolomics has also been applied to examine the change of metabolites after dairy intake in controlled intervention trials. Bertram *et al.* [99] investigated the metabolic response of milk and meat intake in 8-year-old boys for seven days using NMR-based metabolomics and found that milk consumption reduced urinary hippurate excretion and increased serum SCFA level. Both GC-MS and NMR-based serum metabolomics failed to discriminate between intake of probiotic and non-probiotic acidified milk in the subjects aged from 18–79 years, while an increase in lactate, glutamine, proline, creatinine/creatine, aspartic acid, and 3-hydroxybutyrate, as well as a reduced glucose level were observed after the 8-week intervention of both acidified milk drinks [100,101]. Moreover, NMR-based urine metabolomics was applied to elucidate the different responses to casein and whey consumption in 12–15 year old overweight adolescents during a 12-week intervention period and a significant increase in urinary urea excretion was found after casein intake but not after whey intake [102]. Piccolo *et al.* [103] revealed that whey protein supplementation in obese women resulted in a unique plasma metabolic pattern during an 8-week weight loss intervention by GC-MS-based metabolomics and decreased Pro- and Cys-related metabolites, as compared with a gelatin-based protein supplementation. Using an NMR-based metabolomics approach, we found that high dairy intake (4–5 dairy products/day) had a significant impact on urinary metabolite profiles in overweight/obese women (age: 18–60 years old) relative to low dairy intake (0–1 dairy products/day) during a 24-week energy-restricted intervention [unpublished data]. The 24-week intervention with high dairy consumption increased urinary citrate excretion and decreased TMAO levels significantly.

In a crossover study, subjects consumed each diet at different time periods, which means that the influence of individual variation is minimized. Hjerpsted *et al.* [104] applied LC-MS metabolomics on urine to evaluate the difference between cheese and butter consumption in the subjects aged from 22–70 years during a 6-week crossover intervention, yet no separation was obtained by multivariate data analysis. However, by using univariate data analysis, the increases in urinary indoxyl sulfate, xanthurenic acid, tyramine sulfate, 4-hydroxyphenylacetic acid, isovalerylglutamic acid, isovalerylglycine, tiglylglycine, and isobutyrylglycine levels were identified after cheese intake. Intriguingly, in a 2-week crossover study, an NMR-based metabolomics approach based on urine and feces samples was able to successfully discriminate between intake of cheese and milk in 18–50 year old healthy men [105]. Cheese intake resulted in increased urinary prolinebetaine, tyrosine and hippurate as well as fecal butyrate and malonate, while reduced levels of citrate, creatine and creatinine in urine and glycerol in feces.

Yde *et al.* [106] investigated the impact of dairy protein (whey and calcium caseinate) on post-exercise plasma metabolism in young male subjects (age: 28 ± 2 years old) using NMR-based metabolomics. No difference in low-molecular-weight metabolites was found between the two dairy proteins. However, whey protein increased VLDL and reduced LDL, while an increase in both VLDL and LDL levels in plasma was observed after intake of caseinate protein [106].

Furthermore, the postprandial effect of dairy protein intake was assessed by metabolomics. Stanstrup *et al.* [107] used a LC-MS-based metabolomics approach to discriminate between different whey protein fractions consumption on plasma metabolite profiles of obese/non-diabetic subjects (age: 44–74 years old) and found that the levels of amino acids and their derivatives were directly correlated with the composition of whey proteins. Moreover, increased cyclic dipeptides were identified after intake of hydrolysed whey, which is probably associated with its insulinotropic effect [107]. Stanstrup *et al.* [108] also observed that intake of whey isolate caused a postprandial increase in amino acids and a reduction in fatty acids in plasma of obese/non-diabetic subjects (age: 40–68 years old).

**Table 1:** Change of metabolites as determined by metabolomics after intake of dairy products.

Reference	Dairy Product	Design	Subject	*N*	Age	Time	Sample	Technique	Metabolite
**Guertin *et al.* [98]**	Butter	E ^a^	Women (44%)	502	64 ± 5	1 year	Serum	UPLC/GC-MS	Methyl palmitate (16:0)↑; 15:0↑; 10-undecenoate↑
**Bertram *et al.* [99]**	Milk	R ^b^	Boy (100%)	24	8	7 days	Urine, plasma	NMR	Urinary hippurate↑; plasma SCFA↑
**Pedersen *et al.* [100]**	Probiotic/non-probiotic acidified milk	R	Women (74%)	61	19–79	8 weeks	Serum	NMR	Lactate↑; 3-hydroxybutyrate↑
**Pedersen *et al.* [101]**	Probiotic/non-probiotic acidified milk	R	Women (74%)	61	19–79	8 weeks	Serum	GC-MS	Lactate↑; glutamine↑; proline↑; creatinine/creatine↑; aspartic acid↑; glucose↓
**Zheng *et al.* [102]**	Casein, whey, skim milk	R	Overweight adolescents (Girl, 62%)	192	12–15	12 weeks	Urine	NMR	**Casein/skim milk:** urea↑
**Piccolo *et al.* [103]**	Whey	R	Obese women (100%)	27	-	8 weeks	Plasma	GC-MS	**Whey *vs.* gelatin protein:** Pro-/Cys-related metabolites↓
**Zheng *et al.* [104]**	Low or high dairy product	R	Women (100%)	38	18–60	24 weeks	Urine	NMR	**High *vs.* low dairy intake:** citrate↑; TMAO↓
**Hjerpsted *et al.* [105]**	Cheese, butter	C ^c^	Women (43%)	23	22–70	6 weeks	Urine	UPLC-MS	**Cheese:** indoxyl sulfate↑; xanthurenic acid↑; tyramine sulfate↑; 4-hydroxyphenylacetic acid↑; isovalerylglutamic acid↑; isovalerylglycine↑; tiglylglycine↑; isobutyrylglycine↑
**Zheng *et al.* [106]**	Cheese, milk	C	Men (100%)	15	18–50	2 weeks	Urine	NMR	**Cheese *vs.* control:** creatine↓; creatinine↓; prolinebetaine↑; choline↓; TMAO↓; tyrosine↑ **Milk *vs.* control:** citrate↑; prolinebetaine↓; TMAO↓; hippurate↓; urea↑ **Cheese *vs.* milk:** citrate↓; creatine↓; creatinine↓; prolinebetaine↑; tyrosine↑; hippurate↑
**Zheng *et al.* [106]**	Cheese, milk	C	Men (100%)	15	18–50	2 weeks	Feces	NMR	**Cheese *vs.* control:** propionate↑; butyrate↑; malonate↓; fecal lipid↑ **Milk *vs.* control:** propionate↑; acetate↑; glycerol↑; malonate↓; choline↓; fecal lipid↑ **Cheese *vs.* milk:** butyrate↑; malonate↑; glycerol↓
**Yde *et al.* [107]**	Whey, calcium caseinate	C	Men (100%)	12	28 ± 2	Postexercise 70–330 min	Plasma	NMR	**Whey:** VLDL↑; LDL↓ **Caseinate:** VLDL↑; LDL↑
**Stanstrup *et al.* [108]**	Whey isolate (WI), whey hydrolysate (WH), α-lactalbumin, caseinoglycomacropeptide	C	Obese, nondiabetic subjects	11	44–74	Postprandial 1–8 h	Plasma	LC-MS	**WH:** methionine sulfoxide↑; cyclo(Pro-Thr)↑; cyclo(Phe-Val)↑; cyclo(Ile-Val)/cyclo(Leu-Val)↑; β-Asp-Leu↑; pGlu-Pro↑; Cyclo(Ala-Ile)↑; pGlu-Leu↑; pGlu-Val↑; N-phenylacetylmethionine↑; methionine↓; hydroxyphenyllactic acid↓; N-phenylacetylmethionine sulfoxide↑; glutamic acid↑ **WI:** threonine↓; indolelactic acid↑; γ-glutamyl-leucine↑; phenylalanine↑; γ-glutamyl-leucine↑; kynurenine↑		
**Stanstrup *et al.* [109]**	Whey isolate (WI), casein	C	Obese, nondiabetic subjects	11	40–68	Postprandial 1–8 h	Plasma	LC-MS	**WI:** leucine/isoleucine↑; γ-glutamyl-leucine↑; tryptophan↑; isoleucine↑; paracetamol↓; threonine↑; γ-glutamyl-methionine↑; lysine↑; β-hydroxyisobutyric acid↑; methionine↑; γ-glutamyl-valine↑; paracetamol sulfate↓; kynurenine↑; paracetamol glucuronide↓; α-keto-3-methylvaleric acid↑; valine↑; citrulline↑; 3-hydroxy-2-methylbutyric acid↑; glutamic acid↑; propionylcarnitine↑; α-hydroxydecanoic acid↓; lauric acid↓; myristic acid↓; hydroxybutyric acid↑ **Casein:** methionine sulfoxide↑; N-phenylacetyl-methionine↑		

^a^ epidemiologic study; ^b^ randomized study; ^c^ crossover study.

Overall, metabolomics has been applied for qualitative assessment of dairy intake under different experimental conditions, where many factors including individual difference, intervention dose and duration as well as daily diet and physical activity may affect the human metabolome. Thus, care should be taken when comparing the results from different studies. In addition, the potential of these metabolites as biomarkers needs to be further established in other intervention or cohort studies before they can be used as valid markers for quantitatively evaluating dairy consumption and for exploring the association between dairy intake and human health.

## 5. Conclusions

A reliable dairy intake assessment method is essential to better explore the impact of dairy products on human health. Metabolomics as a qualitative assessment tool has shown a potential to discriminate between consumption of different dairy products. Furthermore, metabolomics identified several potential metabolites related to dairy intake, but further validations are required to establish valid biomarkers for quantitative assessment of dairy consumption. Currently the most prominent biomarkers candidates include the 14:1 and 17:1 fatty acids and serum 15:0 cholesteryl esters [83].

Metabolomics could be used as an assessment tool of dairy intake, yet further studies are needed to confirm its usefulness and to expand its application in this research area. In addition, a number of suggestions need to be addressed. Metabolomics studies related to dairy consumption have primarily focused on discriminating between intake of different dairy products, but the quantitative association between metabolite profiles and dairy intake have still not been examined. Since different dairy products have different compositions and therefore may exert different metabolic effects, it would be attractive to investigate different types of dairy products separately when evaluating their consumption. Measuring the composition of dairy products may facilitate identification and analysis of exogenous biomarkers in metabolomics datasets obtained from biological samples. It is suggested to further identify and validate exogenous biomarkers, especially more specific biomarkers, as indicators of dairy intake instead of the FFQ method in the dairy nutrition research. Combined biomarkers could be more reliable and valid for dairy intake assessment than a single marker. Finally, biomarkers need to be validated in clinical trials.
